# Climate change adaptation and mitigation strategies for production forests: Trade-offs, synergies, and uncertainties in biodiversity and ecosystem services delivery in Northern Europe

**DOI:** 10.1007/s13280-023-01909-1

**Published:** 2023-08-17

**Authors:** Adam Felton, Salim Belyazid, Jeannette Eggers, Eva-Maria Nordström, Karin Öhman

**Affiliations:** 1 Southern Swedish Forest Research Centre, Swedish University of Agricultural Sciences, Rörsjöv 1, Box 49, 230 53 Alnarp, Sweden; 2 Department of Physical Geography, Stockholm University, 106 91 Stockholm, Sweden; 3 Division of Forest Planning, Department of Forest Resource Management, Swedish University of Agricultural Sciences, 901 83 Umeå, Sweden

**Keywords:** Biodiversity crisis, Climate mitigation, Ecosystem services, Forest adaptation, Forest conservation, Forest resilience

## Abstract

Climate change adaptation and mitigation strategies (CCAMS) are changes to the management of production forests motivated by the need to mitigate climate change, or adapt production forests to climate change risks. Sweden is employing CCAMS with unclear implications for biodiversity and forest ecosystem services (ES). Here, we synthesized evidence from 51 published scientific reviews, to evaluate the potential implications for biodiversity and a range of provisioning, regulating, and cultural ES, from the adoption of CCAMS relative to standard forestry practice. The CCAMS assessed were the adoption of (i) mixed-species stands, (ii) continuous cover forestry, (iii) altered rotation lengths, (iv) conversion to introduced tree species, (v) logging residue extraction, (vi) stand fertilization, and (vii) altered ditching/draining practices. We highlight the complexity of biodiversity and ES outcomes, identify knowledge gaps, and emphasize the importance of evidence-based decision making and landscape-scale planning when navigating choices involving the widespread adoption of CCAMS.

**Supplementary Information:**

The online version contains supplementary material available at 10.1007/s13280-023-01909-1.

## Introduction

Managing the world’s forests means making decisions, many of which are highly complex, with far-reaching collective consequences for humanity’s future. This is because forests provide us with a breadth of essential ecosystem services (ES), from the provision of wood fibres for building and energy, to the regulation of water resources and creation of environments for recreation and wellbeing (Orsi et al. 2020; Girona et al. 2023). Forest management decisions also determine the extent to which forests continue providing habitat for the approximately 70% of terrestrial biodiversity that depend on these ecosystems for their existence (IUCN 2017). Management choices will also influence the continued health of many forest ecosystems in a world of rapidly changing climates and disturbance regimes, while helping to ensure their capacity to sequester and store around 20% of annual anthropogenic CO_2_ emissions per year (Harris et al. 2021). How forests are managed is thus key to averting the global biodiversity crisis, mitigating climate change, and sustaining the vast range of forest goods and services upon which humanity depends.

In 30% of the world’s forest lands, these decisions result in forests being managed primarily for woody biomass (FAO 2020), often using uniform and intensive approaches to silviculture (Duncker et al. 2012). The most intensive alternatives for production forests are planted forests, which now comprise 7% of total forest area globally (FAO 2020), and over 50% of forest land in some European countries (Payn et al. 2015). Whereas intensive production forestry can provide large amounts of biomass per unit area, the implementation of such forestry over extensive areas can threaten biodiversity (Lindenmayer and Franklin 2002; Eide et al. 2020), limit the ES provided (Gamfeldt et al. 2013; Felipe-Lucia et al. 2018), and increase the vulnerability of stands to climate change associated disturbance (Messier et al. 2021; Triviño et al. 2023). Current trends indicate that global reliance on intensively managed production forests will continue to increase (Warman 2014; Payn et al. 2015), often driven by economic incentives (Puettmann et al. 2015). However, these trends are countered by growing awareness of the biodiversity (e.g. species diversity, functional diversity) and ES benefits (e.g. soil carbon storage, berry production, recreational value) from diversifying silviculture by including a wider variety of less intensive practices (Gamfeldt et al. 2013) that better match natural forest disturbance regimes and tree species composition (Berglund and Kuuluvainen 2021; Messier et al. 2021; Raymond et al. 2023).

In Sweden, the majority of productive forest land area is used for wood production, with approximately 12% either formally or voluntarily protected (Statistics Sweden 2020). Swedish forestry is industrial in scale and intensity, as exhibited by Sweden having one of the highest wood extraction intensities (harvested volume to annual increment) in Europe (Levers et al. 2014), and the fifth largest total planted forest area in the world (Payn et al. 2015). This has enabled Sweden to become the third largest exporter of pulp, paper, and sawn timber (SFIF 2018). Sweden primarily achieves this via the even-aged management of Norway spruce (*Picea abie*s) and Scots pine (*Pinus sylvestris*) stands that are harvested after a rotation length that approximates the maximum economic yield. Although successful in terms of biomass production, the widespread uniformity of current management raises concerns regarding impacts on forest biodiversity (SEPA 2022), the breadth of ecosystem services provided, and forest resilience (Ulmanen et al. 2012). Specifically, concerns have been raised that the widespread simplification of Sweden's forests is limiting their resilience to biotic and abiotic disturbance events which are projected to increase due to anthropogenic climate change (Felton et al. 2010a; Hahn et al. 2021). Additional challenges to forestry practices stem from the desire to enhance the carbon sequestration and storage capacity of forest lands (Heck et al. 2018), which in Sweden is used to advocate the increased use of intensified silvicultural practices (for complexities see Pilli et al. 2022), including fertilization, exotic tree species, and ditching (SFA 2018). Both developments drive substantial changes in how production forests are managed and ES are prioritized (Driscoll et al. 2012, Lindenmayer et al. 2012).

Management alternatives for production forests are thus being proposed in Sweden, based on their potential to serve as climate change adaptation and mitigation strategies (hereafter CCAMS, pronounced ‘C-Cams’) (Felton et al. 2016a). We define CCAMS as changes to production forest management motivated by the need to mitigate climate change, or adapt production forests to climate change associated impacts, risks, and uncertainties (Felton et al. 2016a). CCAMS range from (i) extensive changes to the tree species or diversity of trees grown (e.g. the adoption of introduced tree species or mixtures), (ii) changes to temporal or spatial scale of forestry-related disturbance (e.g. the use of continuous cover forestry, altered rotation lengths, stand fertilization, ditching), and (iii) targeted increases in woody biomass removal (e.g. logging residue extraction). Whereas scientific evidence can often provide guidance regarding the implications of changing management for a selected subset of ES, the extent to which multiple ES can be simultaneously derived from production forest alternatives is less clear. This is especially the case in terms of how such CCAMS contrast in their relative capacity to balance net trade-offs and synergies. Providing relevant insights in this regard requires scientific evaluations encompassing an array of ES, biodiversity, and other considerations derived from specific management alternatives. Evaluations can then be made which contrast these outcomes relative to standard forestry practice within a given biogeographical context. By collating such studies for a range of CCAMS, not only are insights provided regarding the collective trade-offs and synergies of a given intervention, but additional ‘meta’ insights can be gained regarding the comparative capacity of a range of CCAMS to sustain either select ES or a wider breadth of ES simultaneously. These outcomes can thereby provide an evidence-based foundation for informed decisions by forest managers, and policy makers (hereafter “decision makers”).

Here, we synthesized the potential implications for biodiversity and a range of provisioning, regulating, and cultural ES, from the adoption of specific CCAMS that modify or fundamentally change standard forestry practice in Sweden. We used the ES framework to evaluate outcomes, which refers to the benefits people obtain either directly or indirectly from ecosystems (Potschin et al. 2016). Our primary aim is to contrast the broad range of resulting biodiversity and ES implications from different CCAMS to (i) provide evidence-based guidance to decision makers, (ii) discern patterns in trade-offs and synergies, and to (iii) identify key remaining uncertainties regarding trade-offs and synergies in biodiversity and ES delivery from different CCAMS.

## Methods

We searched for scientific articles that reviewed biodiversity and ES outcomes relevant to understanding the implications of modifying or changing standard management of even-aged Norway spruce or Scots pine-dominated stands (reference stand condition; hereafter “reference”) as part of CCAMS. The geographical scope of the study was the northern temperate and boreal regions, with the biogeographical relevance of results to Sweden further targeted by search terms including Norway spruce and Scots pine; tree species native to this region. For standard silvicultural practice in Sweden see Felton et al. (2020). The CCAMS assessed were the adoption of (i) mixed-species stands, (ii) continuous cover/uneven-aged forestry, (iii) altered rotation lengths, (iv) conversion to introduced tree species, (v) logging residue extraction, (vi) stand fertilization, and (vii) altered ditching/draining practices. We do not review all possible CCAMS, but identified these as prominent and distinct examples being considered or applied in Sweden, as motivated below. The adoption of CCAMS was contrasted with the reference in terms of projected changes to forest biodiversity, and ES, specifically provisioning services (biomass production, carbon sequestration), cultural services (recreational values, aesthetics), and regulatory services ameliorating abiotic (wind, fire, drought) and biotic risks (pests, pathogens, large herbivore browsing). We also highlight the implications for lichens, due to their direct relevance to biodiversity and often as forage for Saami reindeer *Rangifer tarandus* herds (Harnesk 2022). Our selection of ES topics targeted issues frequently raised by the many forest stakeholders (production outcomes, damage risk, recreation, biodiversity) (Lodin et al. 2017). As the topics chosen for inclusion are to some extent subjective, our results cannot be used to summarize all the potential trade-offs and synergies derived from the CCAMS. Because of (i) the number of CCAMS addressed, (ii) the variety of biodiversity and ES outcomes considered, and (iii) our goal to detect patterns in synergies and trade-offs in the outcomes that result, we were limited in the extent to which underlying processes could be explored for each topic. Although we do address some of these processes, the reviews we cite understandably pursue these issues in greater depth.

The search for articles was conducted by participating authors from June to September 2022, in Web of Science Core Collection and Scopus, and included review articles published in English (S1: search strings). We also obtained results from colleagues and the reference lists of these articles. In total the search resulted in 1126 review articles (S2: inclusion criteria). Articles were screened in two stages by all co-authors, first by title and abstract and then the remaining 240 articles were screened at the full-text level. Any questions on search methodology or inclusion criteria were discussed following preliminary trial runs with all research group members. The full-text screening resulted in 51 review papers cited below (S3: Compiled results summary), which we also provide as a separate list of articles organized by the CCAMS assessed (S4: Review articles cited).

## Results

### Even-aged mixtures

For our purposes, mixtures are stands involving the even-aged production of two or more tree species, none of which comprises ≥ 70% of stand basal area at final harvest (Felton et al. 2022). We consider the adoption of mixtures as CCAMS due to their expected capacity to mitigate some abiotic and biotic risks affecting monocultures, and the increased adaptive capacity provided by mixtures (Jactel et al. 2017; Messier et al. 2021). We found three reviews that specifically target the implications for either biodiversity (Felton et al. 2010b), or biodiversity and a wide range of ecosystem implications (Felton et al. 2016b; Huuskonen et al. 2021), from employing mixed-species alternatives to Scots pine or Norway spruce monocultures in the Fennoscandia region, as well as six reviews targeted at specific ES (biomass provision, risk regulation, soil carbon stocks). Mixtures consisted of spruce–birch (*Betula* spp.) or spruce–pine tree species combinations, that are intimately mixed (stem by stem). Collective review findings indicate that positive outcomes are expected from mixture adoption for biodiversity, aesthetic, and recreational cultural values (e.g. berry collection, hunting), with additional benefits associated with spruce–birch mixtures for water (reduced inorganic nitrogen & dissolved organic carbon (DOC)) and soil properties (Felton et al. 2016b; Huuskonen et al. 2021). Biodiversity benefits from shifting from spruce monocultures to spruce–birch mixtures (increased species richness and abundance) are expected to extend to bird, vascular plant, lichen, and saproxylic beetle assemblages, with neutral or negative impacts on ground and tree living bryophytes (Felton et al. 2010b).

In terms of provisioning services, biomass production outcomes from mixtures face many uncertainties, due to limited empirical studies, variation in study approaches, differences in the species considered, and wood product evaluated (Felton et al. 2016b). In either regard, spruce stands involving 20% birch had a stem volume production capacity between 90 and 105% of spruce monocultures (80–105% at 50% birch), with variation increasing at higher birch percentages (Huuskonen et al. 2021). Spruce stands including 20% pine provided 95% to 120% of spruce monoculture production capacity (80–125% at 50% pine), with outcomes even more divergent as pine percentage increases (Huuskonen et al. 2021). Earlier reviews from northern Europe similarly highlight the variability of outcomes involving spruce–birch mixtures (Hynynen et al. 2010), leading Huuskonen et al. (2021) to conclude that prevalent mixture combinations in Fennoscandia do not notably alter stem wood yield. With respect to the interpretation of production outcomes, Drössler et al. (2018) in their review of experimental studies in Fennoscandia, Germany, and Poland involving spruce–pine, find that overyielding is relatively common (where growth of a mixed stand was greater than the average of both monocultures), whereas few studies identify transgressive overyielding (mixed-species stand more productive than either monoculture).

In terms of regulatory services, Felton et al. (2016b) and Huuskonen et al. (2021) conclude that mixtures should reduce infection risks to spruce from the pathogen *Heterobasidion* spp*.*; though outcomes depend in-part on site management history, which mixture combination and which species of *Heterobasidion* (e.g. *H. annosum* or *H. parviporum*) are considered (Huuskonen et al. 2021). Mixtures may also reduce infection risks from *Armillaria* spp. and needle colonizing endophytic fungi (Felton et al. 2016b). Likewise, the risk of damage from the highly destructive spruce bark beetle *Ips typographus* is also expected to be reduced in spruce–birch mixtures, as is the risk posed to spruce by the pine weevil *Hylobius abietis* (Felton et al. 2016b). Note, however, that spruce–pine mixtures cannot be expected to reduce pine weevil risk (Felton et al. 2016b). Whether windthrow risk is reduced by either mixture depends on how wind damage is interpreted. If the concern is simply to reduce the proportion of the stand at risk of windthrow, then reducing the prevalence of the less mechanically stable Norway spruce is often advocated (Felton et al. 2016b; Jactel et al. 2017). However, it remains unclear whether any resultant benefits are only proportional to the share of the more stable species, or exhibit additional ‘mixture effects’ (Bauhus et al. 2017; Felton et al. 2022). Whereas we found no Fennoscandian review of mixtures that specifically addressed drought, Jactel et al.’s (2017) review concluded that the response of tree mixtures to drought is highly variable, depending on the tree species composition and environmental conditions.

The implications for browsing damage by browsing cervids from changes to forest management are also complex due in-part to spatial considerations (Wallgren et al. 2013). If the unit is stand level (~ 3 ha in Sweden), and all other variables held constant (e.g. high prevalence of large herbivores, limited landscape-scale forage availability), then the addition of birch or Scots pine to a spruce stand could increase overall browsing damage (Felton et al. 2016b; Huuskonen et al. 2021), due to the fact that birch and pine are often selected over spruce, at least by moose (Månsson et al. 2007). However, if landscape forage increases due to the greater use of these mixtures, then this could reduce landscape-scale browsing pressure (Felton et al. 2016b). Felton et al. (2016b) suggest that fire risk is also difficult to decipher in spruce–birch stands, as results will vary depending on context-specific circumstances (e.g. extent of understory vegetation development). In contrast, spruce–pine mixtures can be expected to increase fire hazards relative to spruce monocultures, due to the lower fuel moisture and higher ignition potentials associated with Scots pine (Felton et al. 2016b). In terms of climate regulation and soil carbon stocks, there is some evidence of mixture benefits within temperate environments, whereby broadleaf additions are associated with increased mineral soil carbon, whereas conifers are associated with higher carbon stocks in forest floor soils (Mayer et al. 2020). Notably, the Jonczak et al. (2020) review of birch tree influences on the soil environment was inconclusive with respect to soil organic carbon outcomes.

A potentially important caveat in mixture biodiversity and ES outcomes is tree spatial arrangement. A recent review by Felton et al. (2022) of spruce–birch mixtures suggests that patch-scale mixtures may benefit forest biodiversity, cervid game, and reduce harvesting costs, whereas intimate mixtures may reduce pest and pathogen damage, and possibly benefit some production outcomes (e.g. wood biomass).

### Continuous cover forestry

Continuous cover forestry (CCF) encompasses a wide range of management alternatives (Pommerening and Murphy 2004). To focus on what is clearly delineated as CCF, and much of the regionally available research (Kuuluvainen et al. 2012), we limit consideration to converting even-aged Norway spruce stands to uneven-aged structurally heterogeneous spruce-dominated forests. CCF usage is motivated in-part by the expectation that its adoption may reduce some risks projected to increase in even-aged Norway spruce stands under climate change (Felton et al. 2016a). These increased risks include damage by storms and bark beetles (Subramanian et al. 2016). We did not find a single review that addresses a wide range of ES from adopting CCF in Fennoscandia, however, 11 reviews did so for a limited subset.

Savilaasko et al. (2021) conducted a meta-analysis of the biodiversity implications of CCF vs. even-aged forestry and concludes that CCF in Fennoscandia hosts more forest-dependent species, and has similar species richness and abundance as natural forests. Specifically, forest lichen species richness was significantly higher in CCF than mature even-aged forest (Savilaakso et al. 2021). An earlier qualitative assessment by Kuuluvainen (2012) likewise suggests that CCF favours late-successional forest species better than even-aged stands, at least in the short term for which empirical studies were available; a finding subsequently reiterated by Ekholm et al. (2023).

In terms of provisioning services, Kuuluvainen et al. (2012) evaluated both empirical and modelling studies to suggest, based on knowledge at that time, that one cannot conclude that CCF provides poorer volume production or economic performance. Subsequently, Lundqvist (2017) in his review of empirical studies assessing Fennoscandian Norway spruce even-aged and full-storied uneven-aged forests emphasizes the importance of the residual standing volume to long-term growth outcomes. Lundqvist (2017) finds that CCF involving the moderate intensity harvesting of the largest trees can result in high sustainable volume growth and large stem volumes, though lower long-term volume growth than even-aged forestry. An important caveat is that early CCF field trials were managed sub-optimally, with more competitive volume growth possible today thanks to increased understanding of the stand dynamics of full-storied forests (Lundqvist 2017). Ekholm et al.’s (2023) review updates these results to conclude that while the majority of simulation studies project lower long-term yield from full-storied CCF, limitations in the available research continue to be an obstacle to determine which silvicultural system is the most productive.

In terms of carbon, a recent global review by Mayer et al. (2020) suggests that less intensive silviculture (e.g. CCF) may reduce the loss of soil C stocks relative to more intensive harvesting. Relatedly, a meta-analysis concludes that intensive harvesting generally increases the susceptibility of northern forest soils to carbon, nitrogen, and phosphorus loss (Hume et al. 2018), and Niemienen et al. (2018) indicate that on drained boreal peatlands in Fennoscandia CCF may maintain water levels at heights that reduce soil greenhouse gas (GHG) emissions. As an additional point, a review by Laiho (2011) suggests that CCF stands were probably superior in terms of carbon sequestration, if its use reduces the harvesting of pulpwood sized trees with limited substitution effects.

With respect to regulatory ES, a review by Nevalainen (2017) concludes that the limited evidence available indicates that mature CCF stands are less susceptible to windthrow than even-aged mature stands (Pukkala et al. 2016; Nevalainen 2017). Heightened risks of windthrow can, however, occur when transitioning from even-aged to CCF (Mason 2002). Nevalainen (2017) also highlights that windthrow can lead to spruce bark beetle outbreaks. In this regard, Nevalainen (2017) suggests that the lower prevalence of older large trees in CCF stands could reduce bark beetle risks by limiting their preferred habitat, and via structural complexity favouring their competitors and predators. However, risks from the six-toothed spruce bark beetle (*Pityogenes chalcographus L.*) may not be reduced by CCF adoption, as this species attacks young and old Norway spruce (Nevalainen 2017). With respect to browsing damage by large herbivores, Nevalainen (2017) did not find relevant targeted studies, but provides some evidence that natural regeneration and a lack of soil scarification reduce risks. We did not identify any studies which addressed the relative susceptibility of CCF versus even-aged Norway spruce stands to fire.

In terms of pathogen risks, Nevalainen (2017) concludes that generations of densely packed spruce with high root contact favours the spread of *Heterobasidion* in Norway spruce CCF, and limits the use of some control strategies, including prescribed burning and stump removal. Nevertheless, risks of root rot can be high in both even-aged and CCF stands (Nevalainen 2017), and neither management type is optimal for addressing “badly infected” stands (Laiho et al. 2011). The risk posed by *Gremmeniella abietina* in CCF stands is complicated, as the shading and suppression may increase the susceptibility of young Norway spruce shoots (Nevalainen 2017), and yet infection rates may be less common in naturally regenerated stands, at least for Scots pine (Kallio et al. 1985). Notably, Nevalainen’s (2017) literature review suggests that spruce seedlings may be more susceptible to drought in even-aged than CCF stands, and that drought can predispose a stand to both bark beetle and root rot.

In terms of cultural services and aesthetic values, Gundersen and Frivold’s (2008) review of public preferences for forest structures in the Nordic countries suggests that CCF stands may be preferred due to their variably sized trees and lack of clear-cuts although silvicultural activities will be more frequent. An additional advantage of CCF is their conduciveness to bilberry production and associated recreation (Laiho et al. 2011).

### Rotation length

The choice of rotation length is central to even-aged forestry and refers to the time period between two final fellings (Roberge et al. 2016). Rotation lengths in Sweden are most often determined by optimizing the land expectation value (Roberge et al. 2016), which is the discounted value of the forest following a series of identical rotations (Faustmann 1849). Therefore, we refer to extended (ERL) or shortened rotation lengths (SRL) as those longer or shorter than this economic optimal for a given site index and tree species. For simplicity, we focus on results for SRL (with ERL implications opposite in response unless otherwise indicated) and emphasize that the effects of rotation length variation are often coupled to and altered by associated changes to thinning regimes. We found only two articles reviewing the effects on either biodiversity or ES from altered rotation lengths (Kivinen et al. 2010; Roberge et al. 2016).

For provisioning services, SRL had negative impacts on wood volume (ERL unclear), mean log diameter, bilberry and mushroom production (ERL unclear), and the availability of shrub, winter pasture (Kivinen et al. 2010), and lichen forage for reindeer and other large herbivores (Kivinen et al. 2010; Roberge et al. 2016). However, some provisioning services are expected to decline with ERL (Roberge et al. 2016) including the availability of forage in clear-cuts and opportunities to change/improve planted tree material. Notably, altering rotation length in either direction diverges from maximum economic yield (Roberge et al. 2016), if changes to stand risks are not considered.

With respect to climate change mitigation, Roberge et al. (2016) find that SRL has negative implications for in-forest carbon storage, as well as potentially reducing substitution effects (ERL unclear) if final felling takes place before the culmination of mean annual increment. Rotation length impacts on soil carbon stocks are considered ambiguous (Roberge et al. 2016). They conclude that if the emphasis is placed on substitution benefits, then the net contribution of modified rotations to climate change mitigation would largely depend on how the average wood volume yield is influenced (Roberge et al. 2016). Questions therefore remain regarding how altered rotation lengths may be optimized to balance the benefits of sequestering and storing CO_2_ in growing forests, with the need to substitute carbon-intensive energy sources and building materials with forest biomass.

With respect to regulatory services, Roberge et al. (2016) find that SRL may help control cambium feeding pests (e.g. spruce bark beetle) and root rot; both of serious economic concern in Sweden. However, SRL can also increase risks from regeneration pests (e.g. pine weevil) and fungal pathogens causing needle cast and shoot dieback (Roberge et al. 2016). Results were unclear with respect to rotation length impacts on the control of defoliating insects and browsing damage by large herbivores. With respect to abiotic risk regulation, SRL may increase fire risks, with additional negative outcomes for forest carbon storage (Roberge et al. 2016). In contrast, SRL should reduce windthrow risks, particularly in Norway spruce stands (Roberge et al. 2016), due to this tree species’ vulnerability to storm damage (Valinger and Fridman 2011).

Roberge et al. (2016) conclude that SRL results in negative impacts on several supporting ES, including hydrological integrity, water quality, and soil nutrients. Likewise, cultural services are expected to worsen with SRL, due to decreased aesthetic and recreational values (unclear ERL), and disturbance impacts to cultural heritage (Roberge et al. 2016). Because the majority of both empirical and modelling studies show that species richness and other biodiversity indicators increase with stand age, SRL is expected to negatively impact on forest biodiversity due to reduced key structures (large trees, dead wood), structural complexity, and landscape heterogeneity (Roberge et al. 2016), with only species dependent on open habitat expected to benefit.

### Logging residue extraction

Logging residue removal (LRE) involves extracting branches, tops, and sometimes stumps after final felling and thinning, with the biomass used for bioenergy production to help reduce fossil fuel reliance (Ranius et al. 2018). We found 11 review articles that addressed the biodiversity and ES implications of LRE. The most frequently addressed review topics were LRE implications for biodiversity, production, and soil carbon. Ranius et al. (2018) is the only review we identified that assessed a broad range of biodiversity and ES implications, including cultural services and non-timber forest products, and they highlight the general paucity of long-term or landscape-scale studies of LRE implications.

With respect to biodiversity impacts, concerns often focus on saproxylic organisms that depend on dead wood (Lassauce et al. 2011). A targeted review by Bouget et al. (2012) suggests LRE has negative effects on saproxylic organisms through habitat loss and fragmentation, as well as causing losses of shelter to wildlife in general. Soil fauna and field vegetation can also be negatively affected by altered soil properties, clearance, and compaction (Bouget et al. 2012). However, because no species live exclusively on harvest residues or stumps (Bouget et al. 2012), several reviews suggest that LRE is unlikely to be problematic for biodiversity unless extraction is intensive, with threatened species potentially affected if conducted over large areas, or in landscapes with high conservation values (de Jong and Dahlberg 2017; Persson and Egnell 2018; Ranius et al. 2018).

In terms of provisioning services, Ranius et al. (2018) suggest that LRE could negatively affect future wood production via the loss of soil nutrients. A review by Wall et al. (2012) surmised that field experiences do not result in consistent effects, and Thiffault et al. (2011) add that uncertainties remain due to the limited time periods for which experimental data are available. For example, in their North American and European review of stump harvesting, Persson and Egnell (2018) conclude that stump removal does not appear to affect timber production, at least for the next forest rotation. An expert assessment concludes that for Swedish conditions, final felling LRE is only expected to negatively impact biomass production if conducted on unsuitable sites (de Jong et al. 2017). De Jong et al. (2017) qualify that whereas LRE from thinning generally reduces forest productivity, stump harvesting effects on production are likely negligible.

With respect to soil carbon, systematic reviews by Hume et al. (2018) and Ranius et al. (2018) find that modelling studies often indicate a negative effect from LRE (i.e. decrease in soil carbon), whereas most empirical studies do not report such impacts. This inconsistency is thought to stem from variation in forest management practices and experimental conditions (e.g. time period assessed) (Ranius et al. 2018; Mayer et al. 2020). For example, Persson and Egnell (2018) find support from model and empirical studies for short-term reductions in soil organic carbon, but long-term experiments (32–39 years) do not detect a decline. Importantly, Cowie et al. (2006) and Mayer et al. (2020) conclude that soil carbon losses related to LRE are negligible compared to the climate benefits in terms of avoided GHG emissions from fossil fuels. In terms of general soil conditions, the review by Hume et al. (2018) finds that residue extraction can negatively affect forest floor mineral soil elemental concentrations emphasizing that as LRE effects on carbon and nitrogen concentrations diminish with time, rotation length decisions are critical to avoiding long-term soil carbon loss.

For regulatory services, several reviews suggest that stump removal can reduce root rot infections during subsequent rotations (Vasaitis et al. 2008; Bouget et al. 2012; Persson and Egnell 2018; Ranius et al. 2018), though this requires that most rot contaminated stumps are extracted (Vasaitis et al. 2008). In addition, Ranius et al. (2018) conclude that stump removal can decrease risks from pine weevil, though the observed effect is smaller than for root rot.

With respect to cultural services and non-wood forest products, Ranius et al. (2018) find few studies addressing berry production, and suggest that the limited evidence available does not indicate that LRE is an important threat to berry availability. However, LRE can reduce food resources for game species and thereby potentially negatively affect their populations’ density or the health of individuals; though as the available evidence is indirect, resultant impacts on reindeer populations remain unclear (Ranius et al. 2018). Furthermore, Ranius et al. (2018) conclude that LRE may be positive for recreational access and landscape aesthetics, if machinery ruts, damage to paths, vegetation, and soils are avoided, and with the exception of stump extraction in the short term.

### Fertilization

Nitrogen (N) fertilization (hereafter ‘fertilization’) is advocated to increase forest carbon sequestration (SFA 2018), and in Sweden involves applying ≥ 1 dose of 150 kgNha^−1^ to stands with relatively deep mesic soils, or moderately fertile sand-silt moraine (Saarsalmi and Mälkönen 2001; Rytter et al. 2016). We found 11 review articles addressing its implications for biodiversity or ES, spanning forest biomass growth, soil carbon, and nitrate leaching.

A key determinant of forest provisioning services is forest growth, and the Hedwall et al. (2014) review identifies several experimental studies showing significant growth increases following fertilization, particularly in middle-aged stands (Saarsalmi and Mälkönen 2001). However, several reviews highlight that most empirical studies are on mineral soils approximately 10 years following fertilization (Nohrstedt 2001; Binkley and Högberg 2016), with some longer-term studies subsequently reporting reduced tree growth (Nohrstedt 2001). Several reviews also highlight that positive growth responses to fertilization may be regulated by other nutrients and water availability (Binkley and Högberg 2016), and that fertilization can induce deficiency in other nutrients (Nohrstedt 2001; Saarsalmi and Mälkönen 2001), and reduce wood density due to the limited formation of thick-walled summer-wood cells (Saarsalmi and Mälkönen 2001).

In terms of soil organic carbon, fertilization has opposing influences (Jandl et al. 2007). The Nave et al. (2009) review finds that fertilization can both increase forest growth, and thereby litterfall, and retard the decomposition rates of soil organic matter. On the other hand, fertilization can increase the nutrient content of fresh litter, therefore stimulating decomposition (Nave et al. 2009). This is reflected in an insignificant effect of fertilization on C storage in the forest floor and significant positive effects on C storage in the mineral soil, with the reverse pattern exhibited by C/N ratios (Nave et al. 2009). Fog’s (1988) review conclusion thus still holds that fertilization has inconclusive impacts on the degradation of soil organic matter.

Evidence of N fertilization effects on biodiversity is more abundant for Flora and Funga than Fauna. Two reviews highlight that fertilization limits the N-philic plant species diversity, notably benefiting herbs and grasses at the expense of bryophytes and dwarf shrubs, with this effect persisting into the second rotation (Binkley and Högberg 2016; Sullivan and Sullivan 2018). Fertilization may indirectly reduce understory species diversity or cover, due to denser resultant canopies that limit understory light levels (Binkley and Högberg 2016). Ekblad et al’s. (2013) review finds that below-ground fertilization may have limited effects on mycorrhizal root colonization, while reducing ectomycorrhizal (EM) mycelia. Fertilization also shifts EM fungal communities from nitrophobic to nitrophilic (Ekblad et al. 2013). Overall, Treseder (2008) concludes that fertilization seems to predominantly reduce microbial biomass, strongly affecting fungi, but bacteria only insignificantly.

The potential for N leaching raises additional biodiversity and environmental concerns. Fertilization with 150 kgNha^−1^ can exceed the N retention capacity of forest soils (Binkley and Högberg 2016), and Saarsalmi and Mälkönen (2001) find that low levels of leaching and considerable albeit transient increases in N concentrations in soil solutions can occur at these levels. The risk of N leaching can, however, be more significant in the absence of tree uptake, which is why the clear-cutting of fertilized forests is associated with leaching events of larger magnitude (Hedwall et al. 2014; Binkley and Högberg 2016).

In terms of regulatory services, it is often hypothesized that fertilization may promote insect herbivory damage, due to enhanced tree nutritional quality (Kytö et al. 1996). Whereas the Kytö et al. (1996) review substantiates this at the individual insect level, the effect remains insignificant at population levels. With respect to browsing damage by cervids, N fertilization appears to increase the nitrogen concentration of Scots pine, with associated increases in moose damage to crop trees (Sullivan and Sullivan 2018).

### Introduced tree species

Introduced tree species are often advocated as a means of enhancing wood production, and thus climate change mitigation (SFA 2018). We found seven reviews that addressed the biodiversity or ES implications of introduced tree species, which primarily assessed issues related to pests, pathogens, invasiveness, and effects on native forest species. Despite our addressing introduced tree species collectively, the biodiversity and ES implications are highly dependent on the tree species considered (Felton et al. 2013).

In Sweden, approximately 2% of forest area is dominated by introduced tree species (Forest Europe 2020), with the vast majority comprised by Lodgepole pine *Pinus contorta* (hereafter “Lodgepole”) (Karlman 2001; Backman and Mårald 2016). The use of Lodgepole is restricted to northern Sweden, where it is planted on sites otherwise occupied by Scots pine (Engelmark et al. 2001). Lodgepole’s higher growth rate is expected to enhance provisioning services related to woody biomass (Kjær et al. 2014). However, both biodiversity and some provisioning services may be negatively affected, as Lodgepole’s denser canopy and thicker litter layer may reduce ground lichen growth, and thus winter forage for reindeer (Kivinen et al. 2010). A review of potential ecological effects by Engelmark et al. (2001) likewise raised ecological concerns regarding its potential to spread beyond stand borders. In terms of regulatory services, Karlman (2001) raised concerns regarding pathogen risks, especially with respect to Lodgepole’s vulnerability to *Gremmeniella abietina*, which caused widespread damage in the 1980s (Karlman 2001). In terms of insect pests, Lidelöw and Björkman’s (2001) review suggests that severe damage to Lodgepole pine was inflicted by the needle feeders *Neodiprion sertifer* and *Anthonomus phyllocola*, whereas one of the costliest pest species in Sweden, the pine weevil, poses similar risk to Lodgepole as to Scots pine.

Other exotic tree species have also been introduced in Sweden, although on a much smaller scale and primarily in southern Sweden (Kjær et al. 2014; Backman and Mårald 2016). Felton et al. (2013) reviewed the biodiversity implication and ecological risks of planting four such species, hybrid larch (*Larix eurolepis/L. marschlinsii*), Douglas fir (*Pseudotsuga menziesii*), Sycamore maple (*Acer pseudoplatanus*), and hybrid aspen (*Populus tremula tremuloides*), on sites otherwise planted with Norway spruce. With respect to provisioning services, all four tree species are grown to fulfil specific forestry requirements and production goals, with hybrid aspen one of the fastest growing broadleaf tree species in Europe (Felton et al. 2013). In terms of biodiversity implications, Felton et al. (2013) reviewed the capacity of introduced tree species to provide habitat to native species, with outcomes ranging from negative biodiversity implications for Douglas fir, and neutral for hybrid larch, to positive for sycamore maple and hybrid aspen. This is due to the higher light levels and broadleaf habitats provided by these two introduced broadleaf tree species (Felton et al. 2013). However, Sycamore maple, hybrid aspen, and Douglas fir (to a lesser extent) also have invasiveness potential, with hybrid aspen posing additional hybridization risks with native aspen *Populus tremula* (Felton et al. 2013). In terms of regulatory services, Felton (2013) concluded that the risk for pests and pathogens ranges from low–medium for Sycamore maple, to medium for hybrid aspen, and medium–high for hybrid larch, whereas it is highly uncertain (low–high) for Douglas fir. In terms of cultural services, the effect of exotic tree species on recreation and aesthetical values can be negative, but also neutral or even positive, depending on the tree species and environment introduced (Gundersen and Frivold 2008).

### Ditching/draining

The use of ditches and drainage to improve conditions for forestry is advocated as a means of increasing forest carbon sequestration (SFA 2018). We found two reviews focussing on the direct effects of draining forest wetlands or peatlands on soil carbon stocks (Trettin et al. 1995), GHG emissions (Trettin et al. 1995; Maljanen et al. 2010), and the export of DOC (Trettin et al. 1995). Trettin et al. (1995) conclude that in general soil carbon stocks are reduced after draining, due to increased decomposition from changes in soil aeration and temperature regimes. The soil carbon stock might, however, increase if below-ground biomass production is sufficiently increased by draining (Trettin et al. 1995). Carbon loss from drainage is either emitted as GHGs, usually CO_2_, or leaked into the water as DOC (Trettin et al. 1995). Maljanen et al. (2010) add that the net exchange of CO_2_ in drained peatland forest depends largely on stand age and climate. Site fertility is a potential additional contributor but few studies have addressed this (Maljanen et al. 2010). Drainage usually decreases the emissions of CH_4_ or may even result in CH_4_ uptake in minero-trophic peatlands (Maljanen et al. 2010). Overall, few empirical studies show possible atmospheric benefits of growing trees on drained organic soils (Maljanen et al. 2010).

With respect to biodiversity, reviews by Tolkkinen et al. (2020) and Johansson et al. (2013) highlight that drainage of peatland forests results in sediment, nutrient, and suspended solids transport to streams, causing brownification and eutrophication of waterways, thereby damaging aquatic habitats. For example, increased sedimentation can decrease the distribution of *Fontinalis* moss and invertebrates, reducing populations of freshwater pearl mussel *Margaritifera margaritifera*, and brown trout *Salmo trutta* (Johansson et al. 2013). In terms of cultural services, Gundersen and Frivold (2008) mention that the drainage of peatlands for forestry has resulted in conflicts due to negative impacts on aesthetics.

Two additional reviews focussed on silvicultural practices conducted on drained peatland forests, and their effects on GHG emissions (Nieminen et al. 2018), and the export of nutrients, sediments, and DOC to water (Nieminen et al. 2017, 2018). These reviews highlight that when a peatland forest is harvested, the water table rises due to reduced tree evapotranspiration. Nieminen et al. (2017) highlight that this is especially severe after clear-cutting and may, depending on soil characteristics, result in the release of nutrients, sediments, and DOC into water courses. Nieminen et al. (2018) suggest, however, that if the high evapotranspiration of mature forest lowers water tables, this can cause heightened GHG emissions from decomposing peat.

## Discussion

Our synthesis highlights both the variety of alternative management strategies that Fennoscandia can pursue in response to climate change, and the complex array of outcomes for forest biodiversity and ES that may result from choosing among them. For the CCAMS assessed, each had its own suite of trade-offs, synergies, and uncertainties (Table 1). The outcomes were not, however, evenly distributed, with the biodiversity and ES outcomes of individual CCAMS spanning the mostly positive to the mostly negative, as well as the implications of some CCAMS being primarily defined by uncertainty (e.g. ditching). For example, relative to spruce monocultures, the adoption of mixtures, CCF, and longer rotations were expected to produce positive outcomes for a broader variety of biodiversity and ES categories than were provided by the other CCAMS considered. In contrast, the adoption of e.g. shortened rotation times was expected to improve outcomes for wind resistance, with species specific implications for pest and pathogen resistance.

**Table 1:** Expected stand-level implications of CCAMS for biodiversity and ES

	Biodiversity	Biomass production	Carbon sequestration/stocks	Recreation/aesthetics	Fire resistance	Drought resistance	Browsing resistance	Insect pest resistance	Pathogen resistance	Wind resistance
Mixture SB, SP	↑**	↕	↕^S^	↑**	↕↓**	?	↓*	↑**^It^/↑*↕^*Ha*^	↑*^H,A^	↑*
CCF	↑**	↕	↑*^S^	↑*	?	?	↑*	↑*^It^	↓*^H^↕^*Ga*^	↑*
Fertilization	↓*	↑*	↕^S^	?	?	?	↓*	↓*	?	?
Extended rotations	↑**	↕	↑*	↕	↑*	?	↕	↓**^It^↑*^Ha^	↓**^H^↑*^Ga, Ls^	↓*
Shortened rotations	↓**	↓**	↓*	↓*	↓*	?	↕	↑**^It^↓*^Ha^	↑**^H^↓*^Ga, Ls^	↑*
Logging residue extraction	↓*	↑**↓*	↕^S^	↕	?	?	?	↑*^Sr, *Ha*^	↑*^Sr^	?
Introduced tree species	↑*↓*	↑*	?	↑*↓*	?	?	?	↑*↓*	↑*↓*	?
Ditching	↓*	↑*	↕^S^	↓*	?	?	?	?	?	?

Outcomes are graded in terms of positive “↑”, negative “↓”, variable/similar “↕”, and unaddressed “?”. For forest disturbances, positive arrows mean improved resistance, whereas negative arrows mean decreased resistance. Confidence levels (i.e. *, **) represent subjectively interpreted levels of support as collated from review articles, here indicated as “limited confidence” (*), and “confident” (**), respectively, for the direction of resultant changes indicated, but were not applied to “variable/similar” nor “unaddressed” outcomes. For mixtures we distinguish the implications of conversion of spruce monocultures to spruce–birch (SB) versus spruce–pine (SP), respectively, if outcomes diverge. For introduced tree species we use two symbols to indicate divergent outcomes that are highly dependent on the tree species considered, for which the relevance of these results is limited to the five introduced tree species considered in the reviews assessed. For logging residue extraction, the superscript ‘Sr’ indicates that outcomes were associated with stump removal. For LRE and biomass production, the positive arrow refers to the increased biomass extracted from the forest, whereas the negative arrow refers to potential impacts on future stand production. If particular pests and pathogens dominated concerns and result outcomes, we acknowledge their importance in a superscript as follows: ^*A*^*Armillaria* spp*.*, ^*Ga*^*Gremmeniella abietina *, ^*H*^*Heterobasidion* spp., ^*Ha*^*Hylobius abietis *, ^*It*^*Ips typographus *, ^*Ls*^*Lophodermium seditiosum *. When carbon sequestration/stock arrows refer primarily to changes to soil carbon, a superscript ‘S’ is added

Although our results highlight the breadth of potential benefits from certain CCAMS, we cannot conclude from this that these alternatives are inherently preferable to others, as this depends on which combination of outcomes are infact prioritized by decision makers. Nevertheless, it is notable that some CCAMS were associated with synergistic outcomes for select combinations of ES. For example, mixtures, CCF, and lengthened rotations have the potential to combine positive biodiversity outcomes, with comparable or in some circumstances even increased biomass production or forest carbon stocks. What this highlights is that there are CCAMS available that could be enlisted to help improve habitat availability, while simultaneously maintaining or increasing the potential contribution of production forests to climate change mitigation. Forest management alternatives that can do so may be vital tools to enlist when attempting to tackle two of the largest challenges facing humanity this century; the mitigation of climate change (IPCC 2022) and averting the biodiversity crisis (Diaz et al. 2019). Importantly, the neutral or increased capacity of CCAMS to produce biomass or sequester carbon was in relation to a reference forest stand condition consisting of intensively managed even-aged monocultures; a highly competitive production forestry approach that has the advantage of over 70 years of investment in Sweden to enhance production efficiency (Lindahl et al. 2017).

Regardless of a forest’s projected capacity to produce biomass, and likewise sequester and store carbon, net resultant outcomes increasingly depend on a stand’s capacity to withstand climate change-related disturbances. In this respect, many forest disturbances are projected to increase in Europe and globally this century, including those due to wind, bark beetle, and fire (Seidl et al. 2014, 2017), and all CCAMS assessed had highly individualistic combinations of resistance to abiotic and biotic risks. Furthermore, some CCAMS, including introduced tree species and ditching, have many remaining unknowns with respect to their resistance to abiotic and biotic risk. Depending on the vulnerability of a site to e.g. fire, or wind, these differences can readily dictate the extent to which stands complete their rotations, and thus which goals for a stand are achieved. As such, the decisions taken when selecting among CCAMS will necessarily be constrained by the adaptive requirements of site-specific risks. Notably, whereas some of the CCAMS had the clear potential to reduce certain disturbance risks, these alternatives were nevertheless unlikely to increase a stand’s biodiversity contribution or climate change mitigation capacity. For example, whereas shortened rotations may increase a stand’s storm resistance, its use is also likely to reduce the stand’s potential contribution to biodiversity, biomass production, and carbon storage, as well as aesthetic and recreational values (Roberge et al. 2016). The unique and distinctive nature of the biodiversity and ES outcomes highlights the importance of not conflating the adoption of CCAMS assessed here with a generic capacity to help mitigate climate change and reduce stand vulnerability to generic disturbances, let alone enhance the biodiversity contribution of production stands.

With respect to biodiversity, it is also important to highlight that even if two CCAMS have the same directional response (e.g. positive, Table 1), this overlap in relative change can obscure potentially large differences in the specific habitats created and species benefited. For example, lengthened rotations can be expected to increase key structural contributors to biodiversity, including the availability of larger/older trees, larger dead wood, and vertical understory complexity; tree species diversity may remain similar (Roberge et al. 2016). In contrast, whereas the inclusion of a broadleaf tree species when adopting mixed-species stands is consistent with Sweden’s goals to increase broadleaf habitats (Felton et al. 2016a), it may not increase the availability of older and larger trees in the landscape. Likewise, the impact of CCAMS adoption on biodiversity can be driven by very distinct processes (Felton et al. 2016a). For example, ericaceous dwarf shrubs play important roles in many ecosystem processes and services in boreal forests, via e.g. their contribution to recreational values (berry picking) (Lindhagen and Bladh 2013), carbon cycles (Hensgens et al. 2020), and food for wildlife (Juvany et al. 2023). Some CCAMS can negatively affect the abundance of ericaceous shrubs via different pathways. Whereas stand fertilization can reduce the cover of ericaceous shrubs via the resultant nitrogen-rich soils and competitive conditions (Binkley and Högberg 2016; Sullivan and Sullivan 2018), shortened rotations can instead reduce coverage by increasing soil disturbance (Roberge et al. 2016). In addition, whereas ericaceous shrub coverage can increase under the higher light levels provided by broadleaf trees in mixtures, these benefits may be overridden if production stand densities are too high (Hedwall et al. 2019).

The driving processes underlying negative biodiversity or ES outcomes have a direct bearing on how CCAMS implementation pathways are navigated in Sweden and elsewhere. This is because all CCAMS had at least one negative biodiversity or ES outcome that may require targeted interventions to address or compensate. For example, there is a range of options for reducing habitat losses incurred by logging residue extraction including (i) avoiding use in landscapes with high conservation values (de Jong and Dahlberg 2017), (ii) retaining logging residues from broadleaf tree species that host more red-listed species (Bouget et al. 2012; de Jong and Dahlberg 2017), and (iii) increasing the number of high stumps and retained trees at harvest, as well as (iv) setting aside more forest area (Ranius et al. 2014; de Jong and Dahlberg 2017). In terms of ES, logging residue extraction may also have negative implications in some contexts for wood production and carbon sequestration, due to reduced soil carbon and nitrogen levels (Ranius et al. 2018). These ES impacts can be compensated for using fertilizers and wood ash (de Jong et al. 2017; Ranius et al. 2018), as well as the use of rotation lengths that allow LRE effects on soils to diminish over time (Hume et al. 2018). Importantly, the choice to compensate with increased rotation lengths or stand fertilization will in-turn have their own corresponding suite of positive and negative impacts for forest biodiversity and ES as highlighted by our results summary (Table 1). Similar considerations are needed if buffer zones and sedimentation pools are used to reduce sedimentation from ditching (Johansson et al. 2013), or if CCF is used on drained peatlands to reduce GHG emissions and impacts on water quality (Nieminen et al. 2018).

The uneven distribution of positive or negative biodiversity and ES responses among the CCAMS assessed can be considered an opportunity, especially if decisions can be taken at landscape scales. At landscape scales, the varied responses among CCAMS allow for their combined use to (i) enhance the net availability of specific habitat features (Felton et al. 2016a), (ii) match their abiotic or biotic resistance to biogeographically relevant climate change vulnerabilities, (iii) help meet increasing societal expectations that production forests provide a diverse range of goods and services (Lindahl et al. 2017), (iv) diversify forestry/risk-spread in response to the uncertainties and altered disturbance regimes of climatic change (Seidl et al. 2018), and thereby (v) help both mitigate climate change and tackle the biodiversity crisis. Landscape planning will of course be vital here, as it is an essential means of combining forest management alternatives to efficiently achieve biodiversity and production goals at landscape and regional scales (Michanek et al. 2018).

In general, our synthesis revealed the breadth of knowledge available regarding the CCAMS considered, as well as the many and substantial knowledge gaps that remain. First and foremost, large uncertainties persist with respect to the implications of ditching for a stand’s vulnerability or resistance to abiotic and biotic disturbances. Whereas empirical studies may be available that tackle at least some of the disturbance implications of ditching, the lack of overarching syntheses nevertheless acts as an obstacle to forest stakeholders and decision makers seeking evidence-based guidance. Perhaps more surprisingly, drought resistance largely lacked consideration in the CCAMS review articles assessed (however, see Jactel et al. 2017; Nevalainen 2017). This is despite the importance of drought as a driver of tree mortality in Europe, and the potential for climate change to increase drought frequency and severity (Senf et al. 2020). We also emphasize that even if review articles did tackle relevant issues, some result outcomes retained substantial uncertainties (i.e. “limited” confidence; Table 1). These uncertainties arose due to limited supportive evidence in general, or variable result outcomes arising from differences in the treatments, environmental conditions, or specific context-specific circumstances compiled by the reviews we assessed. For example, uncertainties in production outcomes from mixtures vs. monocultures can vary depending on the tree species mixed and their respective proportions, site conditions, the wood product desired, and the time period during the rotation considered (Felton et al. 2016b).

### Caveats

Our assessment focussed exclusively on the results of review articles. Although this approach has distinct advantages in terms of the breadth of science that can be synthesized (e.g. Ranius et al. 2023), there are limitations. First, by their very nature review articles benefit from drawing conclusions from multiple studies, but invariably this means being one step behind the latest empirical evidence. For this reason, a lack of reviews addressing a topic may indicate that the research field is not fully developed, but their absence cannot be used (as noted above) to infer the absence of useful empirical studies. Second, when a research field is well developed, multiple reviews by different authors and institutions may address the same topic. Whereas this in itself is beneficial and can lend weight to the resultant conclusions, an important proviso is that reviews overlapping in topic and biogeographical region will also largely overlap in many of the empirical studies reviewed. For this reason, it is important not to conflate the number of reviews addressing an issue, with the amount of supportive empirical evidence underlying their collective findings. Third, the reviews we assessed were based on published studies, which in-turn can selectively filter out empirical findings with non-significant results (the “file drawer” problem; Rosenthal 1979). Review studies themselves may thereby amplify the “file drawer” problem (Arnqvist and Wooster 1995). Whereas quantitative systematic reviews can address these issues using visual and statistical techniques (Simmonds 2015), few of the reviews assessed here were meta-analyses (i.e. Treseder 2008; Nave et al. 2009; Hume et al. 2018; Savilaakso et al. 2021). Each of these caveats should therefore be kept in mind when drawing conclusions from this synthesis.

## Conclusion

CCAMS are being enlisted in Sweden and elsewhere to reduce the vulnerability of production forest stands to climate change and associated disturbances, and/or enhance production forest carbon sequestration and storage capacity. However, our results indicate a suite of additional implications that CCAMS adoption can have for biodiversity, biomass production, and recreational/aesthetics values, as well as highly specific trade-offs and synergies among their respective abiotic and biotic disturbance risks. As regional environmental conditions shift outside their historic range of variability, balancing such trade-offs will be increasingly challenging for many decision makers. Under such circumstances the availability of evidence-based guidance will be a crucial foundation to the effective implementation of CCAMS. Nevertheless, we identified many remaining knowledge gaps for a range of ES outcomes, particularly with respect to the implications of CCAMS for stand drought resistance. We look forward to researchers addressing these knowledge gaps, while also emphasizing the need to develop landscape-scale CCAMS strategies that maximize the potential for synergistic biodiversity and ES outcomes. The choices taken will dictate how competing demands for biodiversity and forest ES are met, sustained, and balanced over the coming century, and ideally will help ensure that the climate change transition does not jeopardize the long-term delivery of important forest values.

### Supplementary Information

Below is the link to the electronic supplementary material.

Supplementary file1 (PDF 943 kb)
