# Publisher Correction: Climate change adaptation and mitigation strategies for production forests: Trade-offs, synergies, and uncertainties in biodiversity and ecosystem services delivery in Northern Europe

**DOI:** 10.1007/s13280-023-01925-1

**Published:** 2023-09-19

**Authors:** Adam Felton, Salim Belyazid, Jeannette Eggers, Eva-Maria Nordström, Karin Öhman

**Affiliations:** 1 Southern Swedish Forest Research Centre, Swedish University of Agricultural Sciences, Rörsjöv 1, Box 49, 230 53 Alnarp, Sweden; 2 Department of Physical Geography, Stockholm University, 106 91 Stockholm, Sweden; 3 Division of Forest Planning, Department of Forest Resource Management, Swedish University of Agricultural Sciences, 901 83 Umeå, Sweden

**Publisher Correction: Ambio** 10.1007/s13280-023-01909-1

In the original publication of the article, the Table 1 was formatted incorrectly during the production process. The correct version of Table 1 is provided in this correction.

**Table 1:** Expected stand-level implications of CCAMS for biodiversity and ES

	Biodiversity	Biomass production	Carbon sequestration/stocks	Recreation/aesthetics	Fire resistance	Drought resistance	Browsing resistance	Insect pest resistance	Pathogen resistance	Wind resistance
Mixture SB, SP	↑**	↕	↕^S^	↑**	↕↓**	?	↓*	↑**^It^/↑*↕^*Ha*^	↑*^H,A^	↑*
CCF	↑**	↕	↑*^S^	↑*	?	?	↑*	↑*^It^	↓*^H^↕^*Ga*^	↑*
Fertilization	↓*	↑*	↕^S^	?	?	?	↓*	↓*	?	?
Extended rotations	↑**	↕	↑*	↕	↑*	?	↕	↓**^It^↑*^Ha^	↓**^H^↑*^Ga, Ls^	↓*
Shortened rotations	↓**	↓**	↓*	↓*	↓*	?	↕	↑**^It^↓*^Ha^	↑**^H^↓*^Ga, Ls^	↑*
Logging residue extraction	↓*	↑**↓*	↕^S^	↕	?	?	?	↑*^Sr, *Ha*^	↑*^Sr^	?
Introduced tree species	↑*↓*	↑*	?	↑*↓*	?	?	?	↑*↓*	↑*↓*	?
Ditching	↓*	↑*	↕^S^	↓*	?	?	?	?	?	?

Outcomes are graded in terms of positive “↑”, negative “↓”, variable/similar “↕”, and unaddressed “?”. For forest disturbances, positive arrows mean improved resistance, whereas negative arrows mean decreased resistance. Confidence levels (i.e. *, **) represent subjectively interpreted levels of support as collated from review articles, here indicated as “limited confidence” (*), and “confident” (**), respectively, for the direction of resultant changes indicated, but were not applied to “variable/similar” nor “unaddressed” outcomes. For mixtures we distinguish the implications of conversion of spruce monocultures to spruce–birch (SB) versus spruce–pine (SP), respectively, if outcomes diverge. For introduced tree species we use two symbols to indicate divergent outcomes that are highly dependent on the tree species considered, for which the relevance of these results is limited to the five introduced tree species considered in the reviews assessed. For logging residue extraction, the superscript ‘Sr’ indicates that outcomes were associated with stump removal. For LRE and biomass production, the positive arrow refers to the increased biomass extracted from the forest, whereas the negative arrow refers to potential impacts on future stand production. If particular pests and pathogens dominated concerns and result outcomes, we acknowledge their importance in a superscript as follows: ^*A*^*Armillaria* spp*.*, ^*Ga*^*Gremmeniella abietina *, ^*H*^*Heterobasidion* spp., ^*Ha*^*Hylobius abietis *, ^*It*^*Ips typographus *, ^*Ls*^*Lophodermium seditiosum *. When carbon sequestration/stock arrows refer primarily to changes to soil carbon, a superscript ‘S’ is added

The original article has been corrected.

