# Supplementary material for: Climate change adaptation and mitigation strategies for production forests: Trade-offs, synergies, and uncertainties in biodiversity and ecosystem services delivery in Northern Europe
Source: Ambio. 2023 Aug 17;53(1):1–16. doi: 10.1007/s13280-023-01909-1 (PMC10692060; doi:10.1007/s13280-023-01909-1)
Supplement: Supplementary file 1 — Supplementary file1 (PDF 943 kb) [file 13280_2023_1909_MOESM1_ESM.pdf]

Ambio

Supplementary information

This supplementary information has not been peer reviewed.

Title: **Climate change adaptation and mitigation strategies for production forests: Trade-offs, synergies and uncertainties in biodiversity and ecosystem services delivery in Northern Europe.**

Authors: Adam Felton, Salim Belyazid, Jeannette Eggers, Eva-Maria Nordström, Karin Öhman

Appendix S1. Search strings used when searching for literature

Search strings were first run in Web of Science before repeating with equivalent versions modified for application in Scopus. Searches were conducted in English using the following steps, with the exact search strings used provided below.

The treatment (CCAMS of interest e.g. mixtures) and control search string (e.g. tree species of interest) were entered as a single search (search **#1**), followed by the specific ES of interest (e.g. biodiversity) (search **#2**). These were combined (**#3** = #1 AND #2), and then restricted to "review" articles using the available option to do so in Web of Science (as such search **#4** = **#3** but review articles only). As the automated "review" restriction missed some known review articles (perhaps due to inconsistencies in journal terminology or categorization), we ran an additional separate "review" search string to catch these articles directly (search **#5**). We then combined search **#5** and **#3** (search string **#6**) to capture relevant reviews missed by search **#4**. We then combined search strings "**#4** OR **#6**" to get all review articles captured by either approach (**#7**). Then each ES search string (including biodiversity) was run separately (**#8, #9, #10**, etc..), with each individual ES search string then combined ("AND") with **#1** and **#7** to capture review articles for a given CCAMS addressing that ES. Then the "OR" term was used to capture review articles covering all of the ES for each CCAMS. In other words, we combined the CCAMS search results, with the tree species "control" results, with the biodiversity or ES results, while restricting search outcomes to review articles, via both the automated function and an additional search string. Results were then exported as RIS files to a unique file within a referencing software (e.g. Endnote), where duplicates were removed.

CCAMS search terms

Mixture search terms

("mix*" or "polyculture*" or "admix*" or "intermix*" or "multi-species" or "multiple species")

CCF search terms

(forest* W/5 "continu* cover*") OR (forest* W/5 "natural* regenerat*") OR (forest* W/5 "multiage*") OR (forest* W/5 "alternativ*") OR (forest* W/5 "common* sens*") OR (forest* W/5 "manage*") OR (forest* W/5 "sustainabl*") OR (regenerat* W/5 "cut*") OR (regenerat* W/5 "fell*") OR (regenerat* W/5 "harvest*") OR (regenerat* W/5 "log*") OR (select* W/5 "cut*") OR (select* W/5 "fell*") OR (select* W/5 "harvest*") OR (select* W/5 "log*") OR (partial* W/5 "cut*") OR (partial* W/5 "fell*") OR (partial* W/5 "harvest*") OR (partial* W/5 "log*") OR (alternat* W/5 "cut*") OR (alternat* W/5 "fell*") OR (alternat* W/5 "harvest*") OR (alternat* W/5 "log*") OR (silvicult* W/5 "continu* cover*") OR (silvicult* W/5 "natural* regenerat*") OR (silvicult* W/5 "multiage*") OR (silvicult* W/5 "alternativ*") OR (silvicult* W/5 "common* sens*") OR (silvicult* W/5 "sustainabl*") OR(retent* W/5 "cut*") OR (retent* W/5 "fell*") OR (retent* W/5 "harvest*") OR (retent* W/5 "log*") OR (conserv* W/5 "cut*") OR (conserv* W/5 "fell*") OR (conserv* W/5 "harvest*") OR (conserv* W/5 "log*") OR (gap* W/5 "cut*") OR (gap* W/5 "fell*") OR (gap* W/5 "harvest*") OR (gap* W/5 "log*") OR (patch* W/5 "cut*") OR (patch* W/5 "fell*") OR (patch* W/5 "harvest*") OR (patch* W/5 "log*") OR (dispers* W/5 "cut*") OR (dispers* W/5 "fell*") OR (dispers* W/5 "harvest*") OR (dispers* W/5 "log*")

Rotation length search terms

(“rotation*” NEAR/5 (“length*” OR “time*” OR “frequen*” OR “period*” OR “short*” OR “long*”)) OR (“harvest*” NEAR/5 (“length*” OR “time*” OR “frequen*” OR “period*” OR “short*” OR “long*”))

Logging residue extraction

(residu* OR slash* OR stump* OR biofuel* OR bio-fuel* OR bio-energ* OR bioenerg* or “whole-tree*” OR “whole tree*”)

Introduced tree species

(“Pinus contorta” OR “lodgepole pine” OR "Pseudotsuga menziesii” OR “Douglas fir” OR “Picea sitchensis” OR “Sitka spruce” OR Larix OR larch OR “Acer pseudoplatanus” OR “Sycamore maple” OR “Populus tremula tremuloides” OR “hybrid aspen” OR (“tree species” NEAR/5 (exot* OR introduc* OR foreign* OR import* OR alien OR nonindigenous OR non-indigenous OR nonnative OR non-native)))

Fertilization

(“Picea abies” OR “Norway spruce*” OR “Pinus Sylvestris” OR “Scots pine*”) AND

(“nitrog*” NEAR/5 “fertili*”)

Ditching

((forest* NEAR/5 (ditch* OR drain* OR trench*)) OR (peat* NEAR/5 (ditch* OR drain* OR trench*)) OR (wetland* NEAR/5 (ditch* OR drain* OR trench*)) OR (land* NEAR/5 (ditch* OR drain* OR trench*)) OR (hydrolog*NEAR/5 (forest* OR peat* OR wetland*)))

Control

(("Picea abies" OR "Norway spruce*" OR "Pinus Sylvestris" OR "Scots pine*") OR ("Swed*" OR "Norw*" OR "Finland*" OR "Finnish*" OR "Denmark*" OR "Danish* " OR "Fenno*" OR "Nordic*" OR "Scand*" OR "*boreal*" OR "north* Europe*")) AND ("clear-cut*" OR "clearcut*" OR "clearfell*" OR "clear-fell*" OR "even-age*" OR "monoculture*")

Additional “review” search

Used for getting around the problem of the “reviews only” button excluding papers that are relevant.

(“review*” OR “synthes*” OR

“summar*”) **Biodiversity and**

Ecosystem services *General*

(“ecosystem*” NEAR/5 “service*”)

Biodiversity

("biolog*" NEAR/5 "divers*") OR ("biodivers*" OR "conserv*" OR "species rich*" OR "red-list*" OR "threat*" OR "bird*" OR "lichen*" OR "epiphyt*" OR "bryophyt*" OR "plant*" OR "mammal*" OR "reptile*" OR "dead-wood*")

Biomass production

(“biomass*” OR “harvest*” OR “timber*”) OR (“wood*” NEAR/5 “product*”) OR (“wood*”

NEAR/5 “harvest*”)

Carbon

(“climate*” NEAR/5 “mitigat*”) OR (“carbon” OR “sequest*”)

Wind

(“storm*” or “wind*”)

Fire

(“fire*” or “burn*”)

Drought

(“drought*” OR “dry*”) OR (“water*” NEAR/5 “lack*”) OR (“water*” NEAR/5 “scarc*”) OR (“water*” NEAR/5 “limit*”) OR (“water*” NEAR/5 “low*”)

Pests

(“pest*” OR “Ips typographus“ OR “Hylobius abietis”) OR (“insect*” NEAR/5 “damage*”) OR (“insect*” NEAR/5 “loss*”) OR (“insect*” NEAR/5 “cost*”)

Pathogens

(“pathogen*” OR “disease*” OR “Heterobasidion” OR “Armillaria” OR “Melampsoridium” OR “Gremmeniella”) OR (“fung*” NEAR/5 “damage*”) OR (“fung*” NEAR/5 “loss*”) OR (“fung*” NEAR/5 “cost*”)

Browsing

(“deer*” OR “ungulate*” OR “large herbivore*” OR “reindeer*” OR “moose*” OR “cervid*” OR “brows*” OR “graz*” OR “alces” OR “capreolus” OR “dama” OR “cervus” OR "roe deer" OR "roe- deer*" OR "red deer*" OR "red-deer*" OR “elk*”) AND (“damage*” OR “cost*” OR “loss*”)

Recreation aesthetics

(“recreat*” OR “aesthetic*” OR “wellbeing*” OR “psycholog*” OR “hunt*” OR “cycl*” OR “walk*” OR “hik*” OR “*touris*”) OR (“berr*” NEAR/5 “pick*”) OR (“berr*” NEAR/5 “harvest*”) OR (“mush*” NEAR/5 “pick*”) OR (“mush*” NEAR/5 “harvest*”)

Appendix 2 Inclusion criteria

1. The study must conduct a review of the field, summarizing, synthesizing, or compiling the different biodiversity or ES outcomes from adopting one of the seven targeted CCAMS, and refer to papers that evaluate these issues. Meta-analysis and narrative syntheses that compile previous studies are included, whereas studies reporting their own novel empirical or modelling findings are not.
2. The study must be relevant to understanding the potential biodiversity or ES implications from modifying or converting Norway spruce or Scots pine dominated even-aged forestry in Fennoscandia, via the i) adoption of mixed-species stands, ii) continuous cover forestry, iii) altered rotations lengths, iv) conversion to introduced tree species, v) extraction of logging residues, vi) stand fertilization, or vii) altered ditching practices. At reviewer discretion, reviews that did not specifically address e.g. Norway spruce or Scots pine conversion to CCAMS, could nevertheless be referred to if they provided outcomes deemed relevant (e.g. large global reviews).
3. The study must consider the implications for forest biodiversity, or any of the targeted ES, namely provisioning services (biomass production, carbon sequestration), cultural services (recreational values, aesthetics), or regulatory services ameliorating abiotic (wind, fire, drought) and biotic risks (pests, pathogens, large herbivore browsing).

Appendix 3 Compiled results summary

	DATE SEARCH CONDUCTED	RECORDS IDENTIFIED FROM WOS AND SCOPUS	REMAINING ARTICLES AFTER TITLE AND ABSTRACT REVIEW	ARTICLES THAT MET ALL INCLUSION CRITERIA
**MIXTURES**	8/4/2022	208	64	9
**CCF**	9/4/2022	359	64	11
**ROTATION LENGTH**	28/4/2022	26	12	2
**LOGGING RESIDUE EXTRACTION**	28/4/2022	56	48	11
**FERTILIZATION**	28/4/2022	256	22	11
**INTRODUCED TREE SPECIES**	26/9/2022	211	24	7
**DITCHING / DRAINAGE**	26/9/2022	10	6	7

Appendix 4 Review articles cited

Mixtures

Bauhus, J., D. I. Forrester, B. Gardiner, H. Jactel, R. Vallejo, and H. Pretzsch. 2017. Ecological stability of mixed-species forests. Pages 337-382 Mixed-Species Forests. Springer.

Drössler, L., E. Agestam, K. Bielak, M. Dudzinska, J. Koricheva, M. Liziniewicz, M. Löf, B. Mason, et al. 2018. Over- and Underyielding in Time and Space in Experiments with Mixed Stands of Scots Pine and Norway Spruce. *Forests* 9: 495.

Felton, A., M. Lindbladh, J. Brunet, and Ö. Fritz. 2010. Replacing coniferous monocultures with mixed-species production stands: An assessment of the potential benefits for forest biodiversity in northern Europe. *Forest Ecology and Management* 260: 939-947.

Felton, A., U. Nilsson, J. Sonesson, A. M. Felton, J.-M. Roberge, T. Ranius, M. Ahlström, J. Bergh, et al. 2016. Replacing monocultures with mixed-species stands: Ecosystem service implications of two production forest alternatives in Sweden. *Ambio* 45: 124-139.

Huuskonen, S., T. Domisch, L. Finér, J. Hantula, J. Hynynen, J. Matala, J. Miina, S. Neuvonen, et al. 2021. What is the potential for replacing monocultures with mixed-species stands to enhance ecosystem services in boreal forests in Fennoscandia? *Forest Ecology and Management* 479: 118558.

Hynynen, J., P. Niemistö, A. Viherä-Aarnio, A. Brunner, S. Hein, and P. Velling. 2010. Silviculture of birch (Betula pendula Roth and Betula pubescens Ehrh.) in northern Europe. *Forestry* 83: 103-119.

Jactel, H., J. Bauhus, J. Boberg, D. Bonal, B. Castagneyrol, B. Gardiner, J. R. Gonzalez-Olabarria, J. Koricheva, et al. 2017. Tree Diversity Drives Forest Stand Resistance to Natural Disturbances. *Current Forestry Reports* 3: 223-243.

Jonczak, J., U. Jankiewicz, M. Kondras, B. Kruczkowska, L. Oktaba, J. Oktaba, I. Olejniczak, E. Pawlowicz, et al. 2020. The influence of birch trees (Betula spp.) on soil environment - A review. *Forest Ecology and Management* 477.

Mayer, M., C. E. Prescott, W. E. Abaker, L. Augusto, L. Cécillon, G. W. Ferreira, J. James, R. Jandl, et al. 2020. Influence of forest management activities on soil organic carbon stocks: A knowledge synthesis. *Forest Ecology and Management* 466: 118127.

CCF

Ekholm, A., L. Lundqvist, E. Petter Axelsson, G. Egnell, J. Hjältén, T. Lundmark, and J. Sjögren. 2023. Long-term yield and biodiversity in stands managed with the selection system and the rotation forestry system: A qualitative review. *Forest Ecology and Management* 537: 120920.

Gundersen, V. S., and L. H. Frivold. 2008. Public preferences for forest structures: a review of quantitative surveys from Finland, Norway and Sweden. *Urban Forestry & Urban Greening* 7: 241-258.

Hume, A. M., H. Y. H. Chen, and A. R. Taylor. 2018. Intensive forest harvesting increases susceptibility of northern forest soils to carbon, nitrogen and phosphorus loss. *Journal of Applied Ecology* 55: 246-255.

Kuuluvainen, T., O. Tahvonen, and T. Aakala. 2012. Even-aged and uneven-aged forest management in boreal fennoscandia: A review. *Ambio* 41: 720-737.

Laiho, O., E. Lähde, and T. Pukkala. 2011. Uneven- vs even-aged management in Finnish boreal forests. *Forestry: An International Journal of Forest Research* 84: 547-556.
Lundqvist, L. 2017. Tamm Review: Selection system reduces long-term volume growth in Fennoscandic uneven-aged Norway spruce forests. *Forest Ecology and Management* 391: 362-375.
Mason, W. 2002. Are irregular stands more windfirm? *Forestry* 75: 347-355.
Mayer, M., C. E. Prescott, W. E. A. Abaker, L. Augusto, L. Cecillon, G. W. D. Ferreira, J. James, R. Jandl, et al. 2020. Tamm Review: Influence of forest management activities on soil organic carbon stocks: A knowledge synthesis. *Forest Ecology and Management* 466.
Nevalainen, S. 2017. Comparison of damage risks in even-and uneven-aged forestry in Finland.
Nieminen, M., H. Hokka, R. Laiho, A. Juutinen, A. Ahtikoski, M. Pearson, S. Kojola, S. Sarkkola, et al. 2018. Could continuous cover forestry be an economically and environmentally feasible management option on drained boreal peatlands? *Forest Ecology and Management* 424: 78-84.
Savilaakso, S., A. Johansson, M. Häkkilä, A. Uusitalo, T. Sandgren, M. Mönkkönen, and P. Puttonen. 2021. What are the effects of even-aged and uneven-aged forest management on boreal forest biodiversity in Fennoscandia and European Russia? A systematic review. *Environmental Evidence* 10: 1.

Rotation length

Kivinen, S., J. Moen, A. Berg, and Å. Eriksson. 2010. Effects of modern forest management on winter grazing resources for reindeer in Sweden. *Ambio* 39: 269-278.
Roberge, J.-M., H. Laudon, C. Björkman, T. Ranius, C. Sandström, A. Felton, A. Sténs, A. Nordin, et al. 2016. Socio-ecological implications of modifying rotation lengths in forestry. *Ambio* 45: 109-123.

Logging residue extraction

Bouget, C., A. Lassauce, and M. Jonsell. 2012. Effects of fuelwood harvesting on biodiversity—a review focused on the situation in Europe. *Canadian Journal of Forest Research* 42: 1421-1432.
Cowie, A. L., P. Smith, and D. Johnson. 2006. Does soil carbon loss in biomass production systems negate the greenhouse benefits of bioenergy? *Mitigation and Adaptation Strategies for Global Change* 11: 979-1002.
de Jong, J., C. Akselsson, G. Egnell, S. Löfgren, and B. A. Olsson. 2017. Realizing the energy potential of forest biomass in Sweden–How much is environmentally sustainable? *Forest Ecology and Management* 383: 3-16.
de Jong, J., and A. Dahlberg. 2017. Impact on species of conservation interest of forest harvesting for bioenergy purposes. *Forest Ecology and Management* 383: 37-48.
Hume, A. M., H. Y. H. Chen, and A. R. Taylor. 2018. Intensive forest harvesting increases susceptibility of northern forest soils to carbon, nitrogen and phosphorus loss. *Journal of Applied Ecology* 55: 246-255.
Mayer, M., C. E. Prescott, W. E. Abaker, L. Augusto, L. Cécillon, G. W. Ferreira, J. James, R. Jandl, et al. 2020. Influence of forest management activities on soil organic carbon stocks: A knowledge synthesis. *Forest Ecology and Management* 466: 118127.

Persson, T., and G. Egnell. 2018. Stump harvesting for bioenergy: A review of climatic and environmental impacts in northern Europe and America. *Wiley Interdisciplinary Reviews-Energy and Environment* 7.
Ranius, T., A. Hämäläinen, G. Egnell, B. Olsson, K. Eklöf, J. Stendahl, J. Rudolphi, A. Sténs, et al. 2018. The effects of logging residue extraction for energy on ecosystem services and biodiversity: A synthesis. *Journal of Environmental Management* 209: 409-425.
Thiffault, E., K. D. Hannam, D. Paré, B. D. Titus, P. W. Hazlett, D. G. Maynard, and S. Brais. 2011. Effects of forest biomass harvesting on soil productivity in boreal and temperate forests—A review. *Environmental Reviews* 19: 278-309.
Vasaitis, R., J. Stenlid, I. M. Thomsen, P. Barklund, and A. Dahlberg. 2008. Stump removal to control root rot in forest stands. A literature study. *Silva Fennica* 42: 457.
Wall, A. 2012. Risk analysis of effects of whole-tree harvesting on site productivity. *Forest Ecology and Management* 282: 175-184.

Fertilization

Binkley, D., and P. Högberg. 2016. Tamm Review: Revisiting the influence of nitrogen deposition on Swedish forests. *Forest Ecology and Management* 368: 222-239.
Ekblad, A., H. Wallander, D. L. Godbold, C. Cruz, D. Johnson, P. Baldrian, R. Björk, D. Epron, et al. 2013. The production and turnover of extramatrical mycelium of ectomycorrhizal fungi in forest soils: role in carbon cycling. *Plant and Soil* 366: 1-27.
Fog, K. 1988. The effect of added nitrogen on the rate of decomposition of organic matter. *Biological Reviews* 63: 433-462.
Hedwall, P.-O., P. Gong, M. Ingerslev, and J. Bergh. 2014. Fertilization in northern forests–biological, economic and environmental constraints and possibilities. *Scandinavian Journal of Forest Research* 29: 301-311.
Jandl, R., M. Lindner, L. Vesterdal, B. Bauwens, R. Baritz, F. Hagedorn, D. W. Johnson, K. Minkkinen, et al. 2007. How strongly can forest management influence soil carbon sequestration? *Geoderma* 137: 253-268.
Kytö, M., P. Niemelä, and S. Larsson. 1996. Insects on trees: population and individual response to fertilization. *Oikos*: 148-159.
Nave, L., E. Vance, C. Swanston, and P. Curtis. 2009. Impacts of elevated N inputs on north temperate forest soil C storage, C/N, and net N-mineralization. *Geoderma* 153: 231-240.
Nohrstedt, H. O. 2001. Response of coniferous forest ecosystems on mineral soils to nutrient additions: A review of Swedish experiences. *Scandinavian Journal of Forest Research* 16: 555-573.
Saarsalmi, A., and E. Mälkönen. 2001. Forest fertilization research in Finland: a literature review. *Scandinavian Journal of Forest Research* 16: 514-535.
Sullivan, T. P., and D. S. Sullivan. 2018. Influence of nitrogen fertilization on abundance and diversity of plants and animals in temperate and boreal forests. *Environmental Reviews* 26: 26-42.
Treseder, K. K. 2008. Nitrogen additions and microbial biomass: A meta-analysis of ecosystem studies. *Ecology Letters* 11: 1111-1120.

Introduced tree species

Engelmark, O., K. Sjoberg, B. Andersson, O. Rosvall, G. I. Agren, W. L. Baker, P. Barklund, C. Bjorkman, et al. 2001. Ecological effects and management aspects of an exotic tree

species: the case of lodgepole pine in Sweden. *Forest Ecology and Management* 141: 3-13.
Felton, A., J. Boberg, C. Björkman, and O. Widenfalk. 2013. Identifying and managing the ecological risks of using introduced tree species in Sweden's production forestry. *Forest Ecology and Management* 307: 165-177.
Gundersen, V. S., and L. H. Frivold. 2008. Public preferences for forest structures: a review of quantitative surveys from Finland, Norway and Sweden. *Urban Forestry & Urban Greening* 7: 241-258.
Karlman, M. 2001. Risks associated with the introduction of Pinus contorta in northern Sweden with respect to pathogens. *Forest Ecology and Management* 141: 97-105.
Kivinen, S., J. Moen, A. Berg, and Å. Eriksson. 2010. Effects of modern forest management on winter grazing resources for reindeer in Sweden. *Ambio* 39: 269-278.
Kjær, E. D., A. Lobo, and T. Myking. 2014. The role of exotic tree species in Nordic forestry. *Scandinavian Journal of Forest Research* 29: 323-332.
Lindelöw, A., and C. Björkman. 2001. Insects on lodgepole pine in Sweden - current knowledge and potential risks. *Forest Ecology and Management* 141: 107-116.

Ditching / Drainage

Gundersen, V. S., and L. H. Frivold. 2008. Public preferences for forest structures: a review of quantitative surveys from Finland, Norway and Sweden. *Urban Forestry & Urban Greening* 7: 241-258.
Johansson, T., J. Hjältén, J. de Jong, and H. von Stedingk. 2013. Environmental considerations from legislation and certification in managed forest stands: A review of their importance for biodiversity. *Forest Ecology and Management* 303: 98-112.
Maljanen, M., B. Sigurdsson, J. Guðmundsson, H. Óskarsson, J. Huttunen, and P. Martikainen. 2010. Greenhouse gas balances of managed peatlands in the Nordic countries–present knowledge and gaps. *Biogeosciences* 7: 2711-2738.
Nieminen, M., H. Hokka, R. Laiho, A. Juutinen, A. Ahtikoski, M. Pearson, S. Kojola, S. Sarkkola, et al. 2018. Could continuous cover forestry be an economically and environmentally feasible management option on drained boreal peatlands? *Forest Ecology and Management* 424: 78-84.
Nieminen, M., S. Sarkkola, and A. Laurén. 2017. Impacts of forest harvesting on nutrient, sediment and dissolved organic carbon exports from drained peatlands: A literature review, synthesis and suggestions for the future. *Forest Ecology and Management* 392: 13-20.
Tolkkinen, M. J., J. Heino, S. H. Ahonen, K. Lehosmaa, and H. Mykrä. 2020. Streams and riparian forests depend on each other: A review with a special focus on microbes. *Forest Ecology and Management* 462: 117962.
Trettin, C. C., M. F. Jurgensen, M. R. Gale, and J. W. McLaughlin. 1995. Soil carbon in northern forested wetlands: Impacts of silvicultural practices. *Carbon forms and functions in forest soils*: 437-461.
